# Validation of Omega Subunit of RNA Polymerase as a Functional Entity

**DOI:** 10.3390/biom10111588

**Published:** 2020-11-23

**Authors:** Unnatiben Rajeshbhai Patel, Sudhanshu Gautam, Dipankar Chatterji

**Affiliations:** Molecular Biophysics Unit, Indian Institute of Science, Bangalore 560012, India; unnatiben@iisc.ac.in (U.R.P.); sudhanshu@iisc.ac.in (S.G.)

**Keywords:** ω-subunit, RNA polymerase, structure, silent mutants, plasticity

## Abstract

The bacterial RNA polymerase (RNAP) is a multi-subunit protein complex (α2ββ’ω σ) containing the smallest subunit, ω. Although identified early in RNAP research, its function remained ambiguous and shrouded with controversy for a considerable period. It was shown before that the protein has a structural role in maintaining the conformation of the largest subunit, β’, and its recruitment in the enzyme assembly. Despite evolutionary conservation of ω and its role in the assembly of RNAP, *E. coli* mutants lacking *rpoZ* (codes for ω) are viable due to the association of the global chaperone protein GroEL with RNAP. To get a better insight into the structure and functional role of ω during transcription, several dominant lethal mutants of ω were isolated. The mutants showed higher binding affinity compared to that of native ω to the α2ββ’ subassembly. We observed that the interaction between α2ββ’ and these lethal mutants is driven by mostly favorable enthalpy and a small but unfavorable negative entropy term. However, during the isolation of these mutants we isolated a silent mutant serendipitously, which showed a lethal phenotype. Silent mutant of a given protein is defined as a protein having the same sequence of amino acids as that of wild type but having mutation in the gene with alteration in base sequence from more frequent code to less frequent one due to codon degeneracy. Eventually, many silent mutants were generated to understand the role of rare codons at various positions in *rpoZ*. We observed that the dominant lethal mutants of ω having either point mutation or silent in nature are more structured in comparison to the native ω. However, the silent code’s position in the reading frame of *rpoZ* plays a role in the structural alteration of the translated protein. This structural alteration in ω makes it more rigid, which affects the plasticity of the interacting domain formed by ω and α2ββ’. Here, we attempted to describe how the conformational flexibility of the ω helps in maintaining the plasticity of the active site of RNA polymerase. The dominant lethal mutant of ω has a suppressor mapped near the catalytic center of the β’ subunit, and it is the same for both types of mutants.

## 1. Introduction

A few decades ago, Tom Silhavy proposed that the α-subunit of *E. coli* RNA polymerase was the “Cinderella”-subunit, often ignored as useless little stepsister by larger subunits [1]. However, the ubiquitous role of α-subunit in transcription regulation soon became apparent, and the name of a useless little sister was no more applicable. This review is aimed at suggesting that the smallest subunit ω indeed received step-sisterly treatment from others, but it is now getting clear that ω has an important function in transcription biology.

## 2. The Transcription Machinery of the Cells

Expression of the genetic information stored in DNA is central to the sustenance of life. Gene expression consists of two major steps: transcription and translation. Transcription is the first step of gene expression, which starts with the synthesis of RNA from DNA by the RNA polymerase (RNAP) enzyme. Transcription is a tightly controlled process, necessitated by the essentiality of precise temporal and spatial gene expression. This exquisitely specific enzyme is multi-subunit machinery. It exists with varying degrees of complexity in all cells; however, it is structurally and functionally similar in all distant life forms. Bacterial RNAP can be considered as the most well-studied member of this family of polymerases. It consists of five main subunits, i.e., two α (RpoA) subunits and one subunit of β (RpoB), and β’ (RpoC), the smallest subunit ω (*RpoZ*) forming the enzyme, α2ββ’ω [2]. Together they facilitate the elongation step of transcription, but they require σ factor (RpoD) to initiate transcription reaction from a specific promoter sequence. A single RNA polymerase primarily mediates the transcription of a bacterial genome. The purified *Escherichia coli* RNA polymerase holoenzyme has a subunit structure of α2ββ’ω σ. Holoenzymes can be reversibly dissociated to yield core enzyme (α2ββ’ω) and σ subunit. Like holoenzymes, the core enzyme is catalytically active, but transcription proceeds non-specifically without promoter recognition ability [3].

## 3. RNA Polymerase Machinery as a Whole

The first indication of RNAP activity in the cell came from Weiss and Gladstone’s mammalian enzyme system in 1959 [4]. A literal explosion of data within a short period of this discovery followed [5,6,7,8,9,10]. Almost immediately, and simultaneously, several laboratories identified similar enzyme activities from various sources, which encompassed bacteria, plants, and animals. These initial investigations also revealed many salient features of RNAP, like dependence on all the four NTPs [11], the requirement of divalent cation [11], the role of DNA as a template in this reaction. The next decade of RNAP research was characterized by a flurry of activity, the main thrust of which was directed at evolving improved methodologies to obtain a pure form of the active protein. It is well beyond the scope of this article to explain all the techniques developed and improvements achieved, but the rapidity and vigor with which progress was made in this direction are well documented. One such study was the development of a protocol by Burgess and Jendrisak to purify bacterial RNA polymerase using Polymin P precipitation and DNA-cellulose chromatography [12]. It revealed that the RNA polymerase is a multi-subunit enzyme complex. The enzyme purified using the above-mentioned protocol showed the presence of 4 bands of varying mobilities in SDS-PAGE as well as on the urea-PAGE. These were designated as β’, β, α, and ω in the order of decreasing weight [13]. However, the ω protein was obtained in such small amounts that it was not considered as a valid subunit of the bacterial RNA polymerase. It did not appear to be necessary for activity, though it was noted that the protein was associated with most enzyme preparations in a stoichiometric ratio.

## 4. The Role of ω Subunit

For a long period, probably until the late 1990s, the constitution of the core bacterial RNAP was considered as α2ββ’. Protocols were designed to remove ω subunit from RNAP purification and improve the purity of the enzyme [14]. Understanding the roles of individual subunits of RNAP is a prerequisite to follow the complex mechanism of transcription. The functional characterization using the biochemical and genetic tool for RNAP has assigned specific roles to the different subunits, except for the smallest subunit, ω, whose role in transcription remained elusive [15,16]. Initial experiments showed that the ω-less RNA polymerase could be reconstituted in vitro from individual subunits [17]. Moreover, concerning the wild-type strain, the ω knockout strain of *Escherichia coli* showed retarded growth but appeared morphologically the same, implying that ω may not be an essential subunit of RNAP [2,18]. What followed was a period of considerable lull as far as the ω subunit was concerned, although the research on RNAP kept accelerating. This period was interspersed with cases where a few interesting observations about this small protein kept cropping up, such as identifying two ω subunits from *Bacillus subtilis*. ω1, which is now called epsilon, and ω2, is a homolog of the ω protein from *E. coli* and other bacteria [19,20]. But the debate on whether ω was a subunit of the enzyme or a protein contaminant remained alive until recent times. Meanwhile, the possibility that ω could still be a subunit of RNAP received a fillip when the work described by Gentry and Burgess [21] showed that the majority of ω protein in *E. coli* cell lysate remained bound to RNAP and a peptide of similar size cross-reacting with anti-ω antibodies was present in several enteric bacteria. Subsequently, two articles were published, which questioned the redundancy of ω being merely an abundant contaminant. First was the convincing identification of β’ subunit as the binding site of ω using cross-linking studies [21].

The second set of elegant experiments utilized the natural phenomenon of transcription activation by protein–protein interaction when one of the interacting proteins was tethered to DNA, and the other is a subunit of RNAP. It was shown that a chimera of ω and the λcI repressor if expressed within a bacterium harboring a λ operator appropriately placed upstream to the promoter, could drive transcription from the promoter [22]. Based on these two results, the indecisiveness of whether the protein was a subunit of RNAP marked a turning point in the research on ω subunit.

The attempts to functionally characterize ω subunit as a part of RNAP in bacteria finally bore fruit when Mukherjee and others [22,23,24,25] provided the first indication of the possible function of ω in RNAP in *E. coli*. They reported that when RNAP was purified from *rpoZ*-null strain of *E. coli*, the enzyme was found to be less than 50% activity compared to that of the wild-type enzyme. Another observation they made was that when the ω-less enzyme was subjected to denaturation using 6M urea, the recovery of the enzyme increased upon the addition of ω during reconstitution. Moreover, they also observed that in the absence of ω, RNAP co-purified with another large protein. It was later identified as GroEL. This led to interesting possibilities in the light of another almost simultaneous observation that β’ was the only subunit of *E. coli* RNAP that was not folded by GroEL in vivo [26]. Association of GroEL with RNAP [27,28] had been previously reported, but the report of Houry et al. suggested a functional role of GroEL, in the folding of all the subunits of RNAP except β’, and in their assembly into the final enzyme [25]. It was further confirmed by Mukherjee et al. that the removal of GroEL completely inactivated the enzyme. These findings taken in combination with the observation that ω cross-linked specifically with the β’, led to the hypothesis that ω carries out a function similar to GroEL aiding to the folding and maintenance of the structure of β’ subunit. Subsequently, Ghosh et al., [29] showed that ω simultaneously binds to the N-terminal and C-terminal regions of β’ and also prevents aggregation of β’ during its renaturation in vitro. However, the most noticeable proof was the X-ray crystal structure of *Thermus aquaticus* core RNAP was solved at 3.3 Å resolution [30]. The structure was able to validate an explicit presence of ω subunit as a part of the core enzyme.

## 5. Functions of ω Subunit in RNAP

**Figure 1:**
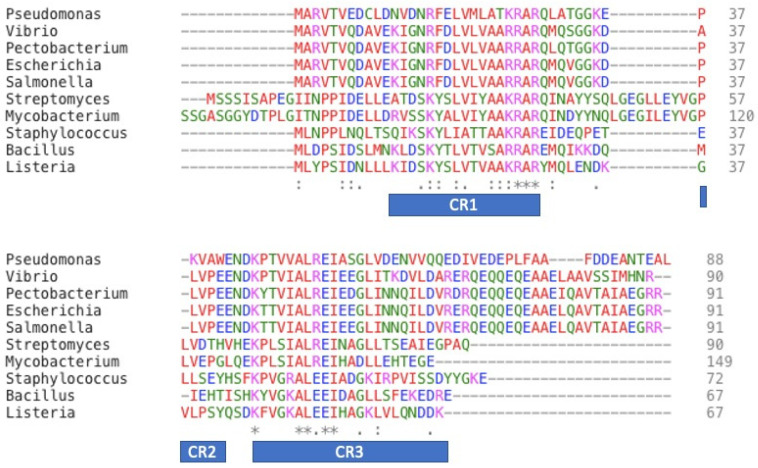
Sequence alignment of Gram-positive and Gram-negative bacterial ω. Identical residues are represented as (*), and similar residues are represented as (: or .). Several residues are highly conserved in both Gram-positive and Gram-negative bacteria like AAKRAR and ALEEI motif. Species names are, in order: *Pseudomonas aeruginosa* (RPOZ_PSEAB), *Vibrio parahaemolyticus* serotype (RPOZ_VIBPA), *Pectobacterium atrosepticum* (RPOZ_PECAS), *Escherichia coli* (RPOZ_ECOLI), *Salmonella typhimurium* (RPOZ_SALTY), *Streptomyces coelicolor* (RPOZ_STRCO), *Mycobacterium tuberculosis* (A0A0T9N9K3_MYCTX), *Staphylococcus aureus* (RPOZ_STAA9), *Bacillus subtilis* (RPOZ_BACSU) and *Listeria monocytogenes* serotype 4a (A0A0E0UX71_LISMM).

The X-ray crystal structure revealed many useful details. In many other prokaryotic organisms, it was observed that ω-subunit plays the role of a molecular latch stabilizing the core enzyme structure and also increases the stability of the core complex [31]. Furthermore, through an extensive analysis of various genome sequences, it was revealed that the subunit homologues to ω exist in archaeal RNAP (subunit RpoK) as well as in eukaryotic RNAPs I, II, and III (subunit RPB6) and their presence in archaea or eukaryotes is essential [31]. When we checked for sequence homology in *rpoZ*, we observed that in gram-positive and in gram-negative bacteria, homologous sequences are present as shown in Figure 1. Detailed sequence alignment revealed that there are three conserved regions, namely, CR1, CR2, and CR3, which are necessary for its function [30].

Further reports supported this notion, when Matthew et al. showed the fragmentation propensity increases for β’-subunit in the absence of ω in *M. smegmatis* [32]. This observation along with the fluorescence experiment [29] mentioned before, showed that ω-subunit has a flexible C-terminal tail that becomes constrained upon binding to β’. Thus, the β’-subunit regained its native conformation upon interacting with ω-subunit.

These studies strengthen the notion that ω is playing a function of the molecular latch to keep the active conformation of the largest subunit. To understand this further, Sarkar et al. employed a toxic mutant screen [33]. They devised a strategy where the entry of GroEL in RNAP was stopped and at the same time a phenotype was ascribed exclusively to ω. Due to the essentiality of GroEL in the living cell, generating GroEL null mutant was not an option. Thus, there was a need for a strategy to thwart the entry of GroEL in RNAP assembly along with mutation in ω. They isolated various dominant lethal mutants and these lethal mutants helped in decoupling the synergy between GroEL and ω. Dominant lethal phenotype means that when expressed in the bacterial cell it had dominant phenotype over wild-type genomic allele. These dominant lethal mutants of ω-subunit isolated in *E. coli* showed a phenomenon known as subunit poisoning by binding to RNA polymerase core. Since the chromosomal deletion of *rpoZ* does not elicit any phenotype, this toxic mutant isolation strategy was used, where a plasmid-based overexpression of the protein had been employed to detect the phenotypic alteration, which is an artificial situation that would elucidate the exclusive role of the smallest subunit of RNAP. The common feature of all these mutants was that they all had more structural elements in the protein as compared to that of the wild-type protein.

**Figure 2:**
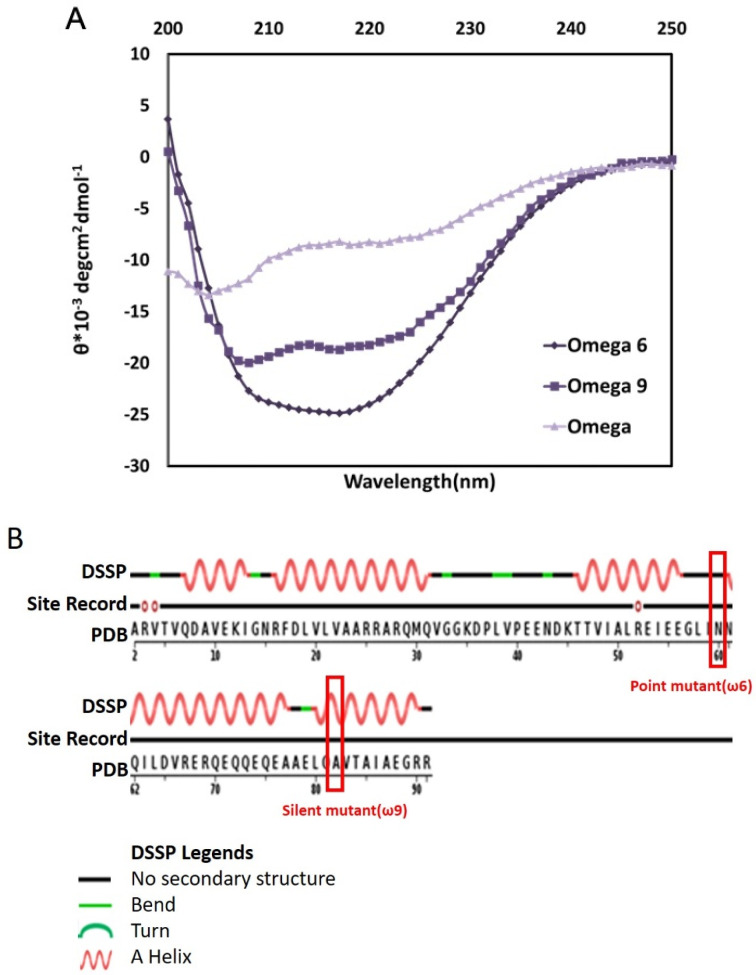
(**A**) Far-UV CD (Far Ultra violet Circular Dichorism) spectra for *E. coli* ω and its lethal mutants, ω_6_, and ω_9_. CD spectra for wild-type protein shows that wild-type ω is less helical in comparison to structured mutants of ω. (**B**). The structure of ω-subunit in RNAP assembly. The ω-subunit exists as a well folded structure in the RNAP assembly in the crystal structure PDB id: 4JKR [35], of *E. coli* RNA polymerase.

Figure 2A shows the far ultra violet circular dichroism (UV CD) spectra for wild-type ω and its dominant lethal mutant ω6 (N60D). The reading at theta_222_ (Θ_222_)shows that the mutant protein is more helical. As reported sometime back [33,34], the change in the structure of the ω subunit due to mutation rendered RNA polymerase defective during initiation of transcription. It should be mentioned here that the ω-subunit exists as a well-folded structure in the crystal structure PDB (id: 4JKR) of *E. coli* RNA polymerase (Figure 2B) [35]. This is one characteristic of intrinsically disordered protein (IDP), where binding coupled folding for the protein takes place upon interaction with its partner/partners [36]. However, we are not sure at this stage whether a protein with only 91 amino acids can be marked as an IDP.

However, this toxic mutant screen led to the serendipitous discovery of a silent mutation in *rpoZ* (GCC to GCT at the 82nd codon of *rpoZ*, frequent to a rare codon for alanine). As mentioned before, the silent mutant ω has the same sequence of amino acids as that of wild-type protein, however, with a third base change in the code for alanine at the 82nd position. The sequence of the mutant ω, having point mutation or silent in nature, was firmly established by mass spectroscopy-based sequence analysis [37]. The silent mutant has a folded conformation, despite having a wild-type sequence (ω9, Figure 2A) and represents a classic case of two conformation of the same protein due to codon degeneracy. We have no experimental proof to pinpoint whether this is due to altered folding rate at the RNA or protein level. However, the silent mutant *rpoZ* or its cognate protein showed dominant lethal phenotype, expectedly. Following this observation, Patel et al. generated many silent mutants to investigate the role of rare codon concerning its position in *rpoZ* gene [34]. Out of those, a few mutants showed a lethal phenotype with concomitant structural alteration. However, most mutants did not show any difference in the growth profile or the secondary structure content. The authors observed that the mutants showing lethal phenotype were mainly mapped close to the C-terminal, falling in the protein’s CR3 region. This region appears to be a sensitive region for mutation, as the other mutants isolated through toxic mutant screen were also mainly mapped in this region.

## 6. Structure–Function Relationship in ω-Subunit

At this stage it was important to evaluate the physical parameters that govern the interaction between mutant or wild-type ω and subassembly α2ββ’ and subsequent recognition of α2ββ’ω with σ-subunit.

**Figure 3:**
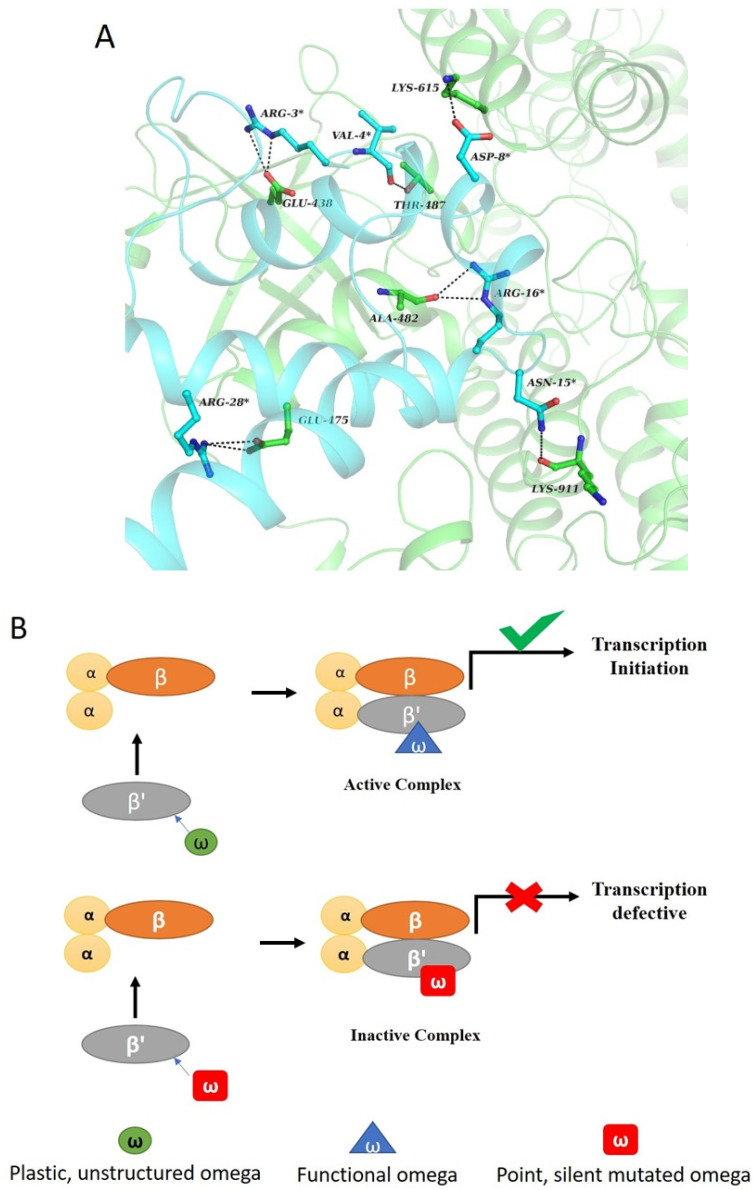
ω, β’ interaction and its schema. (**A**) Interaction between ω and β’ subunit in bacterial RNAP. As shown in the figure, ω (cyan) interacts with β’ (green) via salt bridges and hydrogen bonding (black dotted line) between the side chains. The amino acids of ω interacting with the β’ subunit play an important role in maintaining the structural integrity of the protein. Change in the structural content of ω would interfere with the native interactions. (**B**) ω protein alone is an unstructured protein (green) that undergoes structured transition to ordered structure (blue) upon reconstitution to functional RNAP. Dominant lethal mutant of “ω” (red), point or silent in nature are structured, to begin with which affects the plasticity of the RNAP active site upon reconstitution.

Studies from our laboratory [33,34,38] have shown that dominant lethal mutant of ω- subunit in *E. coli* shows tighter binding with the RNAP (α2ββ’), compared to that of wild-type ω. Thermodynamics of this recognition suggests that the interaction between structured ω and α2ββ’ is driven by high negative enthalpy and a small but unfavorable negative entropy term. The key thermodynamic driving force for the binding reaction is generally a favorable enthalpy contribution. Analysis of crystal structure of RNA polymerase reveals the following details [35]. The interface area of β’ and ω is 1424.0 Å2. There is a significant interaction between both the subunits and any change in ω structure can change the parameters of interaction between the two subunits. Interaction between β’ and ω is guided by virtue of electrostatic interaction between the side chains and hydrogen bonding between the backbone residues (Figure 3A).

The structured ω-subunit binds to α2ββ’ subassembly, which is evident from in vitro reconstitution and surface plasmon resonance (SPR) or isothermal calorimetry (ITC) studies, but the reconstituted enzyme is defective during transcription initiation [33,38]. However, if pre-initiated RNA is supplied to the reaction mixture, elongation of RNA continues unhindered in the presence of the mutated RNAP [33].

This again hints at the importance of the disordered region in ω. As recognized through X-ray structure of RNAP that β’-F-(bridge)-helix and β’-trigger loop encompasses the active center of RNA polymerase and they work in a coordinated fashion to permit the correct placement of NTPs at the active site resulting in their incorporation into the elongating transcripts [39,40,41,42]. Thus, due to the binding of the structured ω, the mobility of the flexible segments at the active site is affected, which manifests into the abrogation of initiation of transcription. The structured ω- affects the plasticity of the active site, which makes the RNAP initiation defective (Figure 3B).

The function of RNAP depends on the movement of many inter and intra-segmental domains relative to each other, and the movement of all domains, including different subunits provides the skeleton of the complex. The major contributors to this structural flexibility of RNAP are the β and β’-subunit as they are the catalytic site of the enzyme [39]. Structural alterations in the catalytic subunit would hinder the molecular motion and would affect the functioning of RNAP. It appears that ω-subunit, whose essential function is to provide a scaffold for one of the catalytic subunits β’ plays a role that affects the movement of the domains at the RNAP active site. This was validated by mapping a suppressor mutant for structured ω, in β’ subunit [33]. This β’ suppressor mutant bears the Y457S substitution located immediately adjacent to the conserved motif NADFDGD, the active site residues on β’-subunit [33,42]. This motif co-ordinate the catalytic Mg+2 ions before the transcription process starts. In an unpublished study from our group, the same suppressor mutant of β’ subunit was used to rescue the effect of silent mutants of ω. It was observed that the same suppressor mutant of β’-subunit, which was able to compensate for a point mutant of ω (N60D), was able to rescue the phenotype due to structured silent mutants of ω as well. It is thus tempting to conclude that the suppressor mutation in β’accommodates the increased structure of ω-subunit and helps in restoring the plasticity of the active site.

## 7. Role of ω-Subunit in Stringent Response

The *rpoZ* gene encoding for ω-subunit in *E. coli*, is located in the same operon as *spoT*. The product of *spoT* gene is responsible for the synthesis and degradation of bacterial alarmone molecules known as ppGpp [43]. Guanosine tetraphosphate (ppGpp) is a bacterial alarmone that enables bacterial cells to swiftly reprogram their gene expression to adapt various stress conditions. ppGpp and its precursor guanosine pentaphosphate (pppGpp), collectively known as (p)ppGpp or magic spot, were discovered more than 50 years ago in *Escherichia coli* during amino acid starvation experiments, and they are known as effectors for stringent response [44]. Although the effect of ppGpp is broadly manifested in bacterial physiology and during regulation of replication and translation, it has a profound effect on transcription rates, like the repression of stable RNA synthesis and the induction of stress response factors and genes that are required for amino acid biosynthesis and transport. Genomic profiling showed that the stringent response alters the expression of hundreds of genes [45,46,47,48,49]. However, the most interesting observation that came from the crystal structure of RNAP with ppGpp, was that the primary site for ppGpp binding is located at β’-ω interface in *E. coli* [35]. When the activity of the enzyme was tested for ppGpp with and without ω-subunit in an in-vitro transcription reaction, it was observed that ω-plays a role in regulating the sensitivity of the enzyme to ppGpp [50,51]. However, it was further debated when the Δ*rpoZ* strain of *E. coli*, showed a normal stationary phase response [52]. Although these observations are contradictory to each other, it can be assumed that there must be a redundancy in the role of ω in the stringent response. The aforementioned statement can be supported with the observation that a second binding site for ppGpp with RNAP is available in the presence of DksA transcription factor [53,54]. This second binding site is more critical to stringent response than the first one [55].

The primary function of ω has been recognized as to provide the scaffold for β’-subunit to fold correctly and to latch it on the α2β subassembly. An earlier study by our group showed that the ω-less RNAP has lower affinity to DNA in comparison to the affinity with native RNAP [25]. It had been reported that in ω-less strain of *E. coli*, the content of the stationary phase sigma factor σS, increased in comparison to that of σ70 [56]. In accordance with the decrease in the amount of RNAP-σ70 holoenzyme, the Δ*rpoZ* strain of *E. coli* showed reduced growth rate at standard growth conditions. However, the slowing down of growth could also be due to the polarity effect on the *spoT* gene located downstream to the *rpoZ* in the same operon. Incorporation of the *rpoZ* gene through homologous recombination experiments randomly at any location, however, rescued the growth rate change negating the effect of *spoT*. There are other observations too worth of consideration. The favored formation of RNAP-σS holoenzyme in the Δ*rpoZ* strain of *E. coli* leads to upregulation of the σS regulon [56]. In addition, studies with chromatin immunoprecipitation experiments showing differential occupancy of ω-less RNAP, which piles up at prophages genes [57], hints at its role in σ-cycling in bacteria. The overexpression of σ70 gene in *E. coli* largely suppresses the Δ*rpoZ* phenotype, further strengthening the notion that the ω-subunit influences selection of the σ factor by the RNAP core.

Several physiological responses in *E. coli*, like biofilm formation, motility, and antibiotic resistance, are dependent on the presence of the *rpoZ* gene [58]. The presence of alternate sigma factors is a hallmark during these responses. Thus, the function of ω-subunit during sigma cycle is worth of further investigation.

We mentioned previously that the enzyme reconstituted with the structured mutant of ω is initiation defective, but it is capable of elongating pre-initiated 11-mer RNA chain, when supplied to the reaction mixture [33]. The only difference between them is the presence of the σ-factor in the initiation complex. Bhowmik et al. showed that the σ-binding to the core was reduced fivefold in the presence of the structured ω-subunit in the core RNAP [38]. As σ-subunit does not bind to ω-subunit directly; it appears this little protein may influence a major event like σ-cycling. The binding domain of ω-subunit to core RNAP near the catalytic center and the very nature of the adjustable conformation of ω prompted us to contemplate this unique role.
